# Differences in clinical importance of Bcl-2 in breast cancer according to hormone receptors status or adjuvant endocrine therapy

**DOI:** 10.1186/s12885-015-1686-y

**Published:** 2015-10-15

**Authors:** Naoko Honma, Rie Horii, Yoshinori Ito, Shigehira Saji, Mamoun Younes, Takuji Iwase, Futoshi Akiyama

**Affiliations:** 1 Department of Pathology, School of Medicine, Toho University, 5-21-16 Omori-Nishi, Ota-ku, Tokyo 143-8540 Japan; 2 Department of Pathology, Cancer Institute, 3-8-31 Ariake, Koto-ku, Tokyo 135-8550 Japan; 3 Breast Medical Oncology, Breast Oncology Center, Cancer Institute Hospital, 3-8-31 Ariake, Koto-ku, Tokyo 135-8550 Japan; 4 Department of Medical Oncology, Fukushima Medical University, School of Medicine, 1 Hikarigaoka, Fukushima City, Fukushima 960-1295 Japan; 5 Department of Pathology and Laboratory Medicine, University of Texas Medical School, 6431 Fannin, MSB 2.270, Houston, TX 77030 USA; 6 Breast Oncology Center, Cancer Institute Hospital, 3-8-31 Ariake, Koto-ku, Tokyo 135-8550 Japan

**Keywords:** Breast cancer, Bcl-2, Triple-negative, Prognosis, Tamoxifen, Estrogen receptor, Progesterone receptor

## Abstract

**Background:**

Bcl-2 plays an anti-apoptotic role, resulting in poor clinical outcome or resistance to therapy in most tumor types expressing Bcl-2. In breast cancer, however, Bcl-2 expression has been reported to be a favorable prognostic factor. The positive correlation of Bcl-2 with estrogen receptor (ER)/progesterone receptor (PR) status, and endocrine therapy frequently given for hormone receptor-positive tumors, may obscure the independent pathobiological role of Bcl-2. We constructed a large systematic study to determine whether Bcl-2 has an independent role in breast cancer.

**Methods:**

Bcl-2 expression was immunohistochemically evaluated and compared with other clinicopathological factors, including clinical outcome, in 1081 breast cancer cases with long follow-up, separately analyzing 634 cases without any adjuvant therapy and 447 cases with tamoxifen monotherapy. The *χ*^2^-test for independence using a contingency table, the Kaplan-Meier method with the log-rank test, and a Cox proportional hazards model were used for the comparison of clinicopathological factors, assessment of clinical outcome, and multivariate analyses, respectively.

**Results:**

In both patient groups, Bcl-2 expression strongly correlated with positive ER/PR status, low grade, negative human epidermal growth factor receptor 2 (HER2) status, and small tumor size, as previously reported. Bcl-2 expression did not independently predict clinical outcome in patients with ER-positive and/or PR-positive tumors or in those who received tamoxifen treatment; however, it was an independent unfavorable prognostic factor in patients with ER-negative/PR-negative or triple-negative (ER-negative/PR-negative/HER2-negative) tumors who received no adjuvant therapy. The latter was even more evident in postmenopausal women: those with hormone receptor-negative or triple-negative tumors lacking Bcl-2 expression showed a favorable outcome.

**Conclusion:**

Bcl-2 expression is an independent poor prognostic factor in patients with hormone receptor-negative or triple-negative breast cancers, especially in the absence of adjuvant therapy, suggesting that the anti-apoptotic effect of Bcl-2 is clearly exhibited under such conditions. The prognostic value of Bcl-2 was more evident in postmenopausal women. The present findings also highlight Bcl-2 as a potential therapeutic target in breast cancers lacking conventional therapeutic targets such as triple-negative tumors. The favorable prognosis previously associated with Bcl-2-positive breast cancer probably reflects the indirect effect of frequently coexpressed hormone receptors and adjuvant endocrine therapy.

## Background

Identification of specific therapeutic targets in cancer tissues is essential to select the most appropriate anti-cancer drugs. In the case of breast cancer, determining estrogen receptor (ER), progesterone receptor (PR), and human epidermal growth factor receptor 2 (HER2) expression has been routine practice for years. Endocrine therapy is considered for patients with hormone receptor-positive (ER-positive and/or PR-positive) tumors, whereas trastuzumab is given to those with HER2-positive tumors. For patients with so-called triple-negative tumors, which are ER-negative/PR-negative/HER2-negative, chemotherapy is the only available treatment. New targets are need to increase treatment options for breast cancer patients, especially with tumors lacking conventional therapeutic targets.

Bcl-2 protein, coded by the bcl-2 gene [1], plays an anti-apoptotic role and inhibits cell death [2], resulting in prolonged cell survival [3]. Bcl-2 is overexpressed in many cancers and contributes to tumor initiation, progression and resistance to therapy [1, 4–8]. There is increasing evidence to suggest that Bcl-2 targeting therapy may be an effective treatment for many cancers [9–15].

Bcl-2 is frequently expressed in normal breast epithelial cells and breast cancer cells, and is known to be upregulated by estrogen [16, 17]. Bcl-2 expression in breast cancer has been reported to positively correlate with differentiated markers or favorable prognostic factors such as ER/PR expression, HER2 negativity, slow proliferation, small tumor size, and so on [18]. Many studies have examined the clinical importance of Bcl-2 expression in breast cancer [19, 20]. In most studies, it has been concluded that Bcl-2 expression predicts a favorable clinical outcome [18, 21–29]. Taking the therapeutic protocol into consideration, Bcl-2 has been reported to be an independent predictor of clinical outcome in patients treated with endocrine therapy [23, 25, 26], but not in those given only local-regional treatment [18, 23, 24, 30]. A favorable clinical outcome in Bcl-2-positive cases is surprising considering the anti-apoptotic nature of Bcl-2; however, correlation of Bcl-2 with differentiated markers seems to be at least partly responsible for these results. Correlation of Bcl-2 expression with ER/PR expression, and endocrine therapy frequently given to patients with hormone receptor-positive tumors, may obscure the independent role of Bcl-2 [31]. In order to elucidate the independent clinicopathological role of Bcl-2 in breast cancer, Bcl-2 expression was assessed immunohistochemically and compared with other clinicopathological factors and with clinical outcome in 1081 breast cancer cases with a long follow-up period. Separate analysis was performed on 634 cases without any adjuvant therapy and 477 cases with adjuvant tamoxifen monotherapy.

## Methods

### Subjects

Among 5763 Japanese patients with primary invasive breast cancer who underwent curative surgery with lymph node dissection at the Cancer Institute Hospital between 1982 and 1993, patients without any adjuvant therapy and with adjuvant tamoxifen monotherapy were selected. Eliminating cases of carcinoma with microinvasion, Stage IV tumors, men, bilateral carcinomas, and no residual carcinoma after biopsy, 634 patients with no adjuvant therapy (11.0 % of the total) and 477 patients with adjuvant tamoxifen monotherapy (8.3 % of the total) were entered into the present study. The follow-up period was 0.5-20.0 years (median 12.8). Grading was performed according to the Japan National Surgical Adjuvant Study of Breast Cancer (NSAS-BC) protocol, which has been confirmed to reflect the prognosis of Japanese breast cancer patients and is routinely used in Japan [32, 33].

Each patient gave informed consent before surgery for the surgical material to be examined for medical purposes. The study protocol was approved by the ethics committee (TB/IRB) of the Cancer Institute.

### Immunohistochemistry

Representative sections of formalin-fixed and paraffin-embedded tissue from archival material as routinely used in the clinical setting were selected for immunohistochemistry. Immunostaining was performed using routine methods on a DAKO Autostainer (Dako, Carpinteria, CA). For ER, PR, and HER2, immunohistochemistry was performed according to the manufacturer’s instructions using an anti-ER mouse monoclonal antibody (clone1D5; Dako), and an anti-PR mouse monoclonal antibody (clone PgR636; Dako), and the HercepTest kit (Dako), respectively. For Bcl-2 examination, an anti-Bcl-2 mouse monoclonal (clone 124; Dako) was used. For antigen retrieval, sections were treated with 98C Target Retrieval Solution pH 9 (Dako) for 40 min. After blocking non-specific activity, the sections were incubated for 30 min at room temperature with anti-Bcl-2 antibody diluted to 1:100. Bound antibodies were detected utilizing the mouse EnVision+, HRP kit (Dako). Appropriate negative and positive controls were included in each batch of immunostains. The results of immunohistochemistry were assessed by two pathologists (N.H. and R.H.) in a blinded fashion, independently examining the whole slide.

**Fig. 1:**
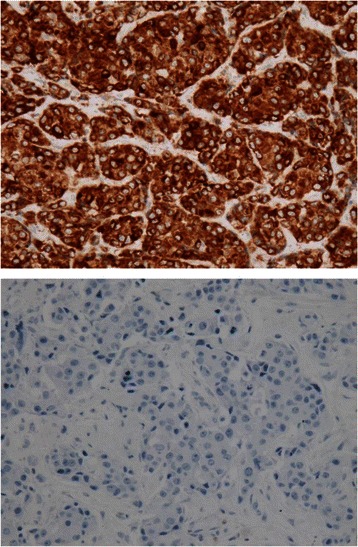
Immunohistochemical images showing typical BCL2 positivity (*upper*) and negativity (*lower*) (immunoperoxidase staining with hematoxylin counterstaining)

Immunoreactivity for ER, PR, and HER2 was estimated according to conventional criteria or the designated procedure as in a previous report [34]. Cytoplasmic immunoreactivity for Bcl-2, shown in Fig. 1, was scored by evaluating the percentage of positively stained cancer cells; the cut-off value for a positive/negative determination was set to 30 % as proposed by others [18, 35]. In most cases, the assessments of the two pathologists were identical, and discrepancies were resolved by joint review of the slides.

### Statistical analysis

The *χ*^2^-test for independence using a contingency table was used to compare Bcl-2 expression with various clinicopathological factors. The Kaplan-Meier method with the log-rank test was used to compare disease-free survival (DFS)/overall survival (OS) according to Bcl-2 expression. The association of various clinicopathological factors with patient outcome was assessed by multivariate analysis using a Cox proportional hazards model. In all instances, the statistical software JMP 8.0 (SAS Institute, Cary, NC) was used. *P* < 0.05, when necessary dividing by the number of factors examined (Bonferroni adjustment), was considered significant.

## Results

### Comparison of Bcl-2 expression with other clinicopathological factors

Bcl-2 positivity was significantly correlated with smaller tumor size, lower grade, ER positivity, PR positivity, and HER2 negativity, in both groups with and without tamoxifen therapy. Premenopausal status at the time of diagnosis correlated with Bcl-2 positivity, yielding a significant result in patients without adjuvant therapy. There was no correlation between Bcl-2 expression and nodal status in either group (Table 1).

**Table 1:** Relation between Bcl-2 status and other clinicopathological factors in patient groups with no adjuvant therapy or tamoxifen monotherapy

	No adjuvant therapy			Tamoxifen monotherapy		
Factors	Bcl-2		*P*-value	Bcl-2		*P*-value
	+ (%)	-		+ (%)	-	
Menopause			0.0049*			0.1249
Post	156 (58)	112		191 (64)	108	
Pre	210 (70)	92		117 (71)	48	
Tumor size			0.0023*			0.0051*
>20 mm	199 (58)	143		178 (61)	112	
≤20 mm	204 (70)	88		138 (74)	49	
Node			0.9049			0.2938
+	96 (63)	56		141 (64)	80	
-	307 (64)	175		175 (68)	81	
Grade			<0.0001*			0.0040*
II + III	244 (54)	205		170 (61)	109	
I	157 (86)	26		145 (74)	52	
ER			<0.0001*			<0.0001*
+	330 (88)	45		290 (74)	100	
-	71 (28)	186		26 (30)	61	
PR			<0.0001*			<0.0001*
+	242 (89)	30		189 (76)	59	
-	159 (44)	200		127 (55)	102	
HER2			<0.0001*			0.0005*
+	19 (21)	70		8 (33)	16	
-	381 (70)	160		308 (68)	145	
Total	634			477		

*Significant, *P* < 0.05/7 = 0.0071 (Bonferroni adjustment)

*Post and Pre* postmenopause and premenopause at the time of diagnosis, + positive, − negative, *ER* estrogen receptor, *PR* progesterone receptor, *HER2* human epidermal growth factor receptor 2

### Survival analysis according to Bcl-2 status in all patients and in subgroups stratified by ER/PR (and HER2) status

**Fig. 2:**
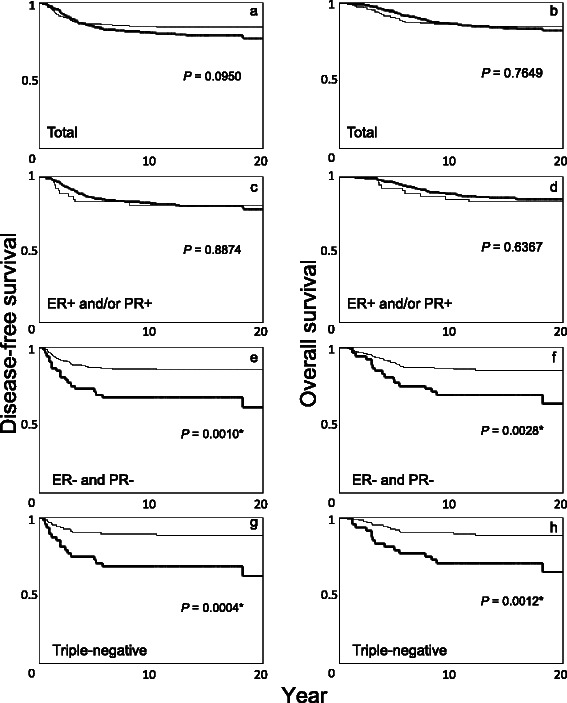
Kaplan-Meier disease-free (**a**, **c**, **e**, **g**) and overall survival (**b**, **d**, **f**, **h**) curves among patients without any adjuvant therapy. Bold lines, Bcl-2-positive; thin lines, Bcl-2-negative. Total patients (**a**, **b**), patients with ER-positive and/or PR-positive tumors (**c**, **d**), patients with ER-negative and PR-negative tumors (**e**, **f**), and patients with triple-negative tumors (**g**, **h**). The *P*-value was determined by the log-rank test. *Significant, *P* <0.05

**Fig. 3:**
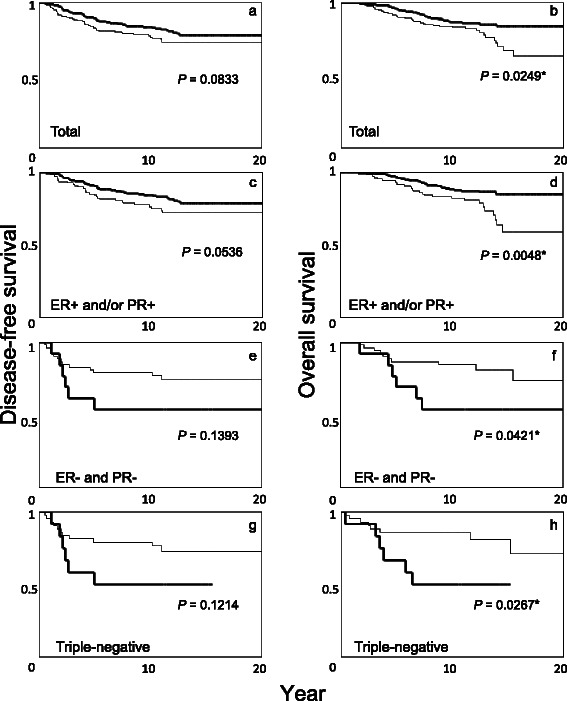
Kaplan-Meier disease-free (**a**, **c**, **e**, **g**) and overall survival (**b**, **d**, **f**, **h**) curves among patients with tamoxifen monotherapy. Bold lines, Bcl-2-positive; thin lines, Bcl-2-negative. Total patients (**a**, **b**), patients with ER-positive and/or PR-positive tumors (**c**, **d**), patients with ER-negative and PR-negative tumors (**e**, **f**), and patients with triple-negative tumors (**g**, **h**). The *P*-value was determined by the log-rank test. *Significant, *P* <0.05

The DFS/OS according to Bcl-2 status in all patients and in subgroups stratified by ER/PR (and HER2) status is shown separately for groups with no adjuvant therapy (Fig. 2) and adjuvant tamoxifen monotherapy (Fig. 3). In patients with no adjuvant therapy, there was no difference in clinical outcome according to Bcl-2 status (Fig. 2a, b) even in the subgroup with ER-positive and/or PR-positive tumors (Fig. 2c, d). By contrast, Bcl-2 positivity was significantly associated with poor clinical outcome in the subgroups with ER-negative and PR-negative tumors (Fig. 2e, f) or with triple-negative tumors (Fig. 2g, h). In patients with adjuvant tamoxifen monotherapy, Bcl-2 positivity was significantly associated with favorable OS (Fig. 3b), including the subgroup with ER-positive and/or PR-positive tumors (Fig. 3d), whereas it was significantly associated with poor OS in the subgroups with ER-negative and PR-negative tumors (Fig. 3f) or triple-negative tumors (Fig. 3h).

### Cox multivariate analysis of recurrence/mortality in invasive breast cancer with no adjuvant therapy or tamoxifen monotherapy

Multivariate analysis was performed taking menopausal status at the time of diagnosis, tumor size, nodal status, grade, ER status, PR status, HER2 status, and Bcl-2 status into consideration in the patient group without adjuvant therapy (Table 2) and in the group with adjuvant tamoxifen monotherapy (Table 3). Bcl-2 positivity independently predicted both recurrence and mortality in the group without adjuvant therapy (Table 2), but not in the group with tamoxifen monotherapy (Table 3). In subgroup analysis considering the ER/PR or HER2 status, Bcl-2 positivity independently predicted recurrence/mortality in ER-negative and PR-negative cases or triple-negative cases with no adjuvant therapy (Table 2), but not in the ER-positive and/or PR-positive subgroup or in the tamoxifen-treated group (Tables 2 and 3).

**Table 2:** Cox multivariate analysis of recurrence/mortality in patients without adjuvant therapy

	Total		ER+ and/or PR+		ER- and PR-		ER-/PR-/HER2-	
	HR (95% CI)	*P*-value	HR (95% CI)	*P*-value	HR (95% CI)	*P*-value	HR (95% CI)	*P*-value
Recurrence								
PostM vs. PreM	0.893 (0.598-1.329)	0.5774	1.167 (0.721-1.887)	0.5268	0.813 (0.419-1.548)	0.5300	0.866 (0.378-1.921)	0.7248
T (>2cm vs. ≤2cm)	0.887 (0.601-1.315)	0.5488	0.895 (0.548-1.460)	0.6573	0.900 (0.469-1.796)	0.7572	0.823 (0.388-1.823)	0.6212
LN (+ vs. -)	2.933 (1.978-4.323)	<0.0001*	2.451 (1.488-4.018)	0.0005*	3.879 (1.973-7.414)	0.0002*	3.522 (1.484-7.793)	0.0056*
Grade (II+III vs. I)	2.121 (1.294-3.649)	0.0024*	2.182 (1.274-3.921)	0.0039*	2.056 (0.600-12.900)	0.2844	1.947 (0.560-12.290)	0.3309
ER (+ vs. -)	0.723 (0.444-1.193)	0.2026						
PR (+ vs. -)	0.588 (0.370-0.931)	0.0235*						
HER2 (+ vs. -)	1.313 (0.720-2.282)	0.3617	0.947 (0.227-2.660)	0.9276	1.563 (0.754-3.138)	0.2237		
Bcl-2 (+ vs. -)	2.544 (1.512-4.351)	0.0004*	1.568 (0.759-3.802)	0.2398	3.369 (1.681-6.755)	0.0007*	3.321 (1.556-7.231)	0.0021*
Mortality								
PostM vs. PreM	0.914 (0.592-1.407)	0.6829	1.252 (0.721-2.174)	0.4219	0.807 (0.418-1.529)	0.5137	0.877 (0.385-1.928)	0.7455
T (>2cm vs. ≤2cm)	1.008 (0.657-1.563)	0.9705	1.098 (0.628-1.933)	0.7436	0.917 (0.470-1.872)	0.8058	0.862 (0.399-1.965)	0.7139
LN (+ vs. -)	3.186 (2.082-4.853	<0.0001*	2.701 (1.532-4.769)	0.0007*	3.749 (1.907-7.178)	0.0002*	3.425 (1.444-7.576)	0.0065*
Grade (II+III vs. I)	2.431 (1.364-4.672)	0.0020*	2.577 (1.358-5.316)	0.0031*	1.871 (0.547-11.728)	0.3575	1.795 (0.519-11.302)	0.3963
ER (+ vs. -)	0.602 (0.354-1.032)	0.0646						
PR (+ vs. -)	0.534 (0.316-0.894)	0.0169*						
HER2 (+ vs. -)	1.406 (0.762-2.485)	0.2667	1.237 (0.293-3.557)	0.7363	1.653 (0.795-3.337)	0.1742		
Bcl-2 (+ vs. -)	2.399 (1.385-4.205)	0.0017*	1.222 (0.578-3.004)	0.6210	3.143 (1.559-6.306)	0.0016*	3.081 (1.432-6.719)	0.0043*

Factors considered other than Bcl-2 is menopausal status, tumor size, nodal status, grade, ER, PR and HER2 status

*ER* estrogen receptor, *PR* progesterone receptor, *HER2* human epidermal growth factor receptor 2, *HR* hazard ratio, *PostM and PreM* postmenopause and premenopause at the time of diagnosis, *T* tumor size, *LN* lymph node, *+* positive; − negative

*Significant, *P* < 0.05.

**Table 3:** Cox multivariate analysis of recurrence/mortality in patients with tamoxifen monotherapy

	Total		ER+ and/or PR+		ER- and PR-		ER-/PR-/HER2-	
	HR (95% CI)	*P*-value	HR (95% CI)	*P*-value	HR (95% CI)	*P*-value	HR (95% CI)	*P*-value
Recurrence								
PostM vs. PreM	1.048 (0.671-1.665)	0.8404	1.154 (0.728-1.879)	0.5482	1.464 (0.561-4.045)	0.4367	1.523 (0.542-4.585)	0.4255
T (>2cm vs. ≤2cm)	1.921 (1.232-3.097)	0.0035*	1.637 (1.016-2.726)	0.0426	4.859 (1.334-31.224)	0.0137*	4.370 (1.165-28.412)	0.0268
LN (+ vs. -)	3.004 (1.953-4.736)	<0.0001*	3.466 (2.118-5.898)	<0.0001*	1.851 (0.664-5.160)	0.2342	2.438 (0.822-7.620)	0.1075
Grade (II+III vs. I)	1.810 (1.165-2.887)	0.0079*	1.902 (1.184-3.147)	0.0074*	1.296 (0.414-4.933)	0.6696	1.447 (0.454-5.593)	0.5464
ER (+ vs. -)	1.128 (0.634-2.092)	0.6890						
PR (+ vs. -)	0.770 (0.501-1.183)	0.2328						
HER2 (+ vs. -)	1.162 (0.437-2.577)	0.7395	2.002 (0.591-5.117)	0.2347	0.518 (0.080-1.954)	0.3613		
Bcl-2 (+ vs. -)	0.929 (0.604-1.449)	0.7430	0.892 (0.561-1.450)	0.6377	1.745 (0.576-4.804)	0.3078	1.958 (0.621-5.682)	0.2387
Mortality								
PostM vs. PreM	1.084 (0.659-1.822)	0.7532	1.482 (0.863-2.646)	0.1568	0.991 (0.335-2.923)	0.9871	0.869 (0.257-2.898)	0.8166
T (>2cm vs. ≤2cm)	2.498 (1.468-4.506)	0.0005*	2.114 (1.198-3.951)	0.0090*	8.196 (1.545-152.292)	0.0093*	10.872 (1.682-234.909)	0.0081*
LN (+ vs. -)	2.861 (1.768-4.778)	<0.0001*	2.997 (1.733-5.430)	<0.0001*	1.826 (0.565-6.013)	0.3097	3.070 (0.845-12.889)	0.0888
Grade (II+III vs. I)	1.849 (1.118-3.168)	0.0161*	2.184 (1.258-3.969)	0.0051*	1.079 (0.312-4.332)	0.9076	1.299 (0.367-5.344)	0.6924
ER (+ vs. -)	1.354 (0.714-2.696)	0.3614						
PR (+ vs. -)	0.564 (0.342-0.920)	0.0219*						
HER2 (+ vs. -)	1.188 (0.440-2.697)	0.7099	1.968 (0.577-5.095)	0.2486	0.536 (0.074-2.341)	0.4345		
Bcl-2 (+ vs. -)	0.801 (0.496-1.307)	0.3695	0.688 (0.415-1.161)	0.1591	2.550 (0.797-7.752)	0.1106	3.443 (0.999-11.894)	0.0502

Factors considered other than Bcl-2 were menopausal status, tumor size, nodal status, grade, ER, PR and HER2 status

*ER* estrogen receptor, *PR* progesterone receptor; *HER2* human epidermal growth factor receptor 2, *HR* hazard ratio, *PostM and PreM* postmenopause and premenopause at the time of diagnosis, *T* tumor size, *LN* lymph node, *+* positive, − negative

*Significant, *P* <0.05.

### Survival analysis according to Bcl-2 status or Cox multivariate analysis of recurrence/mortality in ER-negative and PR-negative cases or triple-negative cases with no adjuvant therapy stratified by menopausal status

**Fig. 4:**
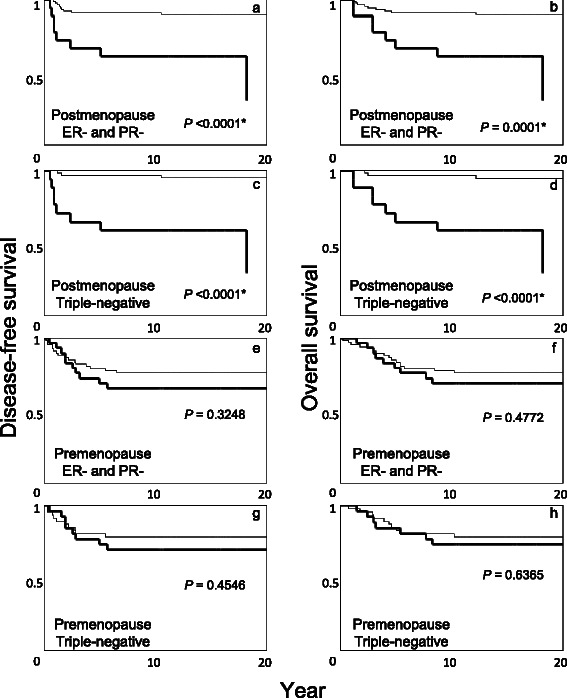
Kaplan-Meier disease-free (**a**, **c**, **e**, **g**) and overall survival (**b**, **d**, **f**, **h**) curves in ER-negative and PR-negative cases or triple-negative cases without any adjuvant therapy stratified by menopausal status at diagnosis. Bold lines, Bcl-2-positive; thin lines, Bcl-2-negative. Postmenopausal patients with ER-negative and PR-negative tumors (**a**, **b**), postmenopausal patients with triple-negative tumors (**c, d**), premenopausal patients with ER-negative and PR-negative tumors (**e**, **f**), and premenopausal patients with triple-negative tumors (**g**, **h**). The *P*-value was determined by the log-rank test. *Significant, *P* <0.05

Among ER-negative and PR-negative cases or triple-negative cases with no adjuvant therapy, survival analysis according to Bcl-2 status or Cox multivariate analysis of recurrence/mortality was performed, stratifying by the menopausal status. In survival analysis, Bcl-2-positive cases exhibited significantly poorer DFS/OS in postmenopausal, but not in premenopausal, women (Fig. 4). Bcl-2 remained an independent predictor of recurrence/mortality in postmenopausal, but not in premenopausal, women in the ER-negative and PR-negative group in multivariate analyses, including tumor size, nodal status, grade, and HER-2 status; the hazard ratio of Bcl-2 (positive vs. negative) was 17.591 (95 % CI, 4.942 to 75.462; *P* <0.0001) for recurrence and 9.587 (95 % CI, 3.066 to 32.493; *P* <0.0001) for mortality in postmenopausal women, but 1.801 (95 % CI, 0.753 to 4.188; *P* = 0.1810) for recurrence and 1.667 (95 % CI, 0.672 to 3.970; *P* = 0.2612) for mortality in premenopausal women. Also, in multivariate analyses of triple-negative cases, including tumor size, nodal status, and grade, Bcl-2 positivity independently predicted recurrence/mortality in postmenopausal, but not in premenopausal, women; the hazard ratio of Bcl-2 (positive vs. negative) was 18.607 (95 % CI, 4.685 to 103.022; *P* <0.0001) for recurrence and 12.391 (95 % CI, 3.501 to 57.617; *P* <0.0001) for mortality in postmenopausal women, but 1.434 (95 % CI, 0.545 to 3.653; *P* = 0.4540) for recurrence and 1.283 (95 % CI, 0.464 to 3.354; *P* = 0.6170) for mortality in premenopausal women.

## Discussion

The clinicopathological role of Bcl-2 in breast cancer was systematically investigated in 634 cases without any adjuvant therapy and 447 cases with tamoxifen monotherapy using full sections from routinely processed archival materials as used in the clinical setting. As others reported [18, 31], Bcl-2 expression was positively correlated with ER and PR expression, but negatively correlated with HER2 expression, grade, and tumor size, confirming Bcl-2’s association with favorable prognostic factors or differentiated markers in both groups with and without tamoxifen (Table 1). In patients who did not receive adjuvant therapy, Bcl-2 expression significantly correlated with premenopausal status (Table 1). Correlation of Bcl-2 expression with premenopausal status has been previously reported by Zhang et al. [36], whereas Hellmans et al. found no relation [23]. Higher premenopausal serum estrogen may promote Bcl-2 expression through ER [37], although this may be offset by the commonly observed correlation of ER expression with postmenopausal status [38], leading to inconsistent results.

In the tamoxifen-treated group, Bcl-2 positivity correlated with better OS in overall patients and in the subgroup with ER-positive and/or PR-positive tumors, but with poor OS in the subgroups with ER-negative and PR-negative tumors or with triple-negative tumors (Fig. 3); however, Bcl-2 was not an independent predictor of clinical outcome in overall patients or subgroups when ER/PR or HER2 status is taken into consideration (Table 3). In the group without adjuvant therapy, there was no evidence that Bcl-2 positivity was a favorable prognostic factor in the entire group or in the subgroup with ER-positive and/or PR-positive tumors (Fig. 2). The finding that a favorable prognosis with Bcl-2 positivity was more evident in the tamoxifen-treated group than in the no adjuvant group is consistent with other reports [18, 21–26, 31]. The favorable prognosis reported for Bcl-2-positive tumors therefore seems to at least partly reflect the indirect effect of coexpressed hormone receptors.

In the group of patients that did not receive adjuvant therapy, Bcl-2 positivity significantly correlated with poor clinical outcome in patients with hormone receptor-negative (ER-negative and PR-negative) or triple-negative tumors (Fig. 2). In multivariate analysis, Bcl-2 positivity independently predicted recurrence/mortality in the entire group of patients and in hormone receptor-negative or triple-negative cases, but not in ER-positive and/or PR-positive cases (Table 2). It seems that the anti-apoptotic effect of Bcl-2, which usually correlates with poor clinical outcome or resistance to therapy in tumors other than breast cancer [5, 7, 8], is evident only in cases without hormone receptors and without adjuvant therapy. In the tamoxifen-treated group, Bcl-2 positivity was also significantly correlated with poor OS among patients with hormone receptor-negative or triple-negative tumors, but that did not reach statistical significance in DFS, probably due to the small number of cases in that group (Fig. 3). Only a few studies have examined the clinical importance of Bcl-2 in subgroups considering the status of hormone receptors or HER2. Tawfik et al. reported that Bcl-2 expression was an independent poor prognostic factor in 124 triple-negative breast cancers; however, multivariate analysis in that study did not include tumor size or nodal status, which are the most powerful prognostic factors. Further, information about adjuvant therapy was not described in that study [39]. Ryu et al. did not find Bcl-2 useful in predicting clinical outcome in 94 triple-negative cancers [40]. In a study by Dawson et al., the hazard ratio for Bcl-2 positive vs. negative expression was reportedly larger in ER-negative, PR-negative, and triple-negative than ER-positive, PR-positive, and non-triple-negative cancers, respectively, which is in line with our present study; however, adjuvant therapy was not considered in each comparison [28].

Interestingly, the prognostic value of Bcl-2 in hormone receptor-negative or triple-negative cases without any adjuvant therapy was more evident in postmenopausal, but diminished in premenopausal, women (Fig. 4). In other words, postmenopausal patients with Bcl-2-negative and hormone receptor-negative (or triple-negative) cancers exhibited quite favorable clinical outcome even without adjuvant chemotherapy (Fig. 4a-d). The reason is not known; however, the present results suggest the need for a reevaluation of adjuvant chemotherapy for these patients.

In the present study, a 30 % cut-off was used to determine Bcl-2 status, as proposed by Silvestrini et al., who examined the outcome predictive power of Bcl-2 status using several cut-offs, and showed that a 30–40 % cut-off yielded the best result; however, a 10 % cut-off has been more frequently used by others [20]. We obtained similar results using a 10 % cut-off for Bcl-2, but the outcome predictive power decreased compared with the Bcl-2 status determined by a 30 % cut-off (data not shown). The reason for this is not known, but the usefulness of a 30 % cut-off for Bcl-2 status as reported in our study seems to validate Silvestrini’s results. Since the overall results were essentially the same irrespective of whether the cut-off value was 10 % or 30 %, it is unlikely that the different cut-off value we used in this study is the reason why our results are different from studies showing that bcl-2 positivity is a predictor of favorable outcome.

Bcl-2 antisense therapy has been suggested for various tumors in an *in vitro* setting [11–13]. The development of Bcl-2 inhibitors has been explored [41, 42], and small-molecule inhibitors of Bcl-2 such as ABT–737 and ABT-199 have recently been introduced [10, 15]. These Bcl-2 inhibitors were shown to be effective in prolonging survival in animal models bearing lymphoid malignancies [10, 14]. Emerging evidence also suggests the usefulness of this type of therapy in breast cancer [9, 43, 44]. ABT-199 has been reported to improve the response of ER-positive tumors to tamoxifen [43, 44]. Oakes et al. reported that ABT–737 sensitized primary basal-like breast cancers with elevated Bcl-2 levels to docetaxel and improved response and OS in an *in vivo* setting, suggesting that elevated Bcl-2 expression constitutes a predictive response marker in breast cancer [9]. This recently demonstrated usefulness of Bcl-2 inhibitors in basal-like breast cancers expressing Bcl-2, together with our present finding that Bcl-2 positivity is correlated with poor clinical outcome in patients with hormone receptor-negative or triple-negative tumors, suggest that Bcl-2-targeted therapy may improve the poor clinical outcome of patients with such tumors expressing Bcl-2. This evidence warrants a clinical study of Bcl-2-targeted therapy in breast cancer. Bcl-2 examination is expected to improve prediction of the clinical outcome or to predict response to Bcl-2-targeted therapy in breast cancer.

## Conclusions

Bcl-2 expression is an independent poor prognostic factor in patients with hormone receptor-negative or triple-negative breast cancers, especially in the absence of adjuvant therapy, suggesting that the anti-apoptotic nature of Bcl-2 is clearly exhibited under such conditions. The prognostic value of Bcl-2 is more evident in postmenopausal women. The favorable prognosis previously observed in Bcl-2-positive cancer seems to reflect the indirect effect of frequently coexpressed hormone receptors and adjuvant endocrine therapy.

## Abbreviations

ER: Estrogen receptor
PR: Progesterone receptor
HER2: Human epidermal growth factor receptor 2
DFS: Disease-free survival
OS: Overall survival
CI: Confidence intervals
